# Quality of Life and Pain after Proximal Humeral Fractures in the Elderly: A Systematic Review

**DOI:** 10.3390/medicina59101728

**Published:** 2023-09-27

**Authors:** Janette Iking, Karen Fischhuber, Josef Stolberg-Stolberg, Michael J. Raschke, Jan Christoph Katthagen, Jeanette Köppe

**Affiliations:** 1 Department of Trauma, Hand and Reconstructive Surgery, University Hospital Muenster, Albert-Schweitzer-Campus 1, Building W1, 48149 Muenster, Germany; janette.iking@ukmuenster.de (J.I.);; 2 Research Group “Mathematical Surgery”, University Hospital Muenster, University of Muenster, 48149 Muenster, Germany; 3 Institute of Biostatistics and Clinical Research, University of Muenster, Schmeddingstrasse 56, 48149 Muenster, Germany

**Keywords:** proximal humeral fractures, quality of life, locking plate fixation, reverse shoulder arthroplasty

## Abstract

*Background and Objectives:* The proximal humeral fracture (PHF) is one of the most common fractures in elderly patients. A PHF might influence the quality of life (QoL) on several different levels, especially in elderly patients, but it is unclear which treatment option results in a better QoL outcome. Therefore, we aimed to systematically review the current literature for studies that have analyzed the QoL and pain of elderly patients treated either surgically or non-operatively for PHF. *Materials and Methods:* A comprehensive search of the literature was performed in the PubMed database from January to April 2023. Studies describing the QoL or the level of pain of patients older than 60 years with the EuroQoL-5 Dimension (EQ-5D) score or the visual analogue scale (VAS) after the treatment of PHF, either non-operatively (non-OP), with open-reduction and internal fixation using a locking plate (LPF), or with reverse total shoulder arthroplasty (RTSA) were included. Twelve studies were analyzed descriptively and the individual risk of bias was assessed using the ROB2 and ROBINS-I tools. *Results:* A total of 12 studies with 712 patients at baseline were included (78% female sex, mean age 75.2 years). The reported VAS scores at 12-month follow-up (FU) ranged from 0.7 to 2.5. The calculated overall mean VAS score across all studies showed a decreasing tendency for all treatments, with an increasing FU time up to 12 months after PHF. None of the studies reported any significant differences of the EQ-5D across the groups. The overall calculated EQ-5D indices showed an increasing trend after 6–8 weeks FU, but did not differ significantly between the three treatments. *Conclusions:* In conclusion, the current literature suggests that there are no clinically important differences between the QoL or pain in elderly patients with PHF after non-operative treatment or surgical treatment with LPF or RTSA. However, the number of studies and level of evidence is rather low and further trials are urgently needed.

## 1. Introduction

The proximal humeral fracture (PHF) is one of the most common fractures in elderly patients, especially in women [1]. Incidences will increase due to ongoing demographic changes [1,2,3,4,5,6]. Depending on several factors, such as the complexity of the fracture or the age of the patient, non-operative or surgical treatment options are recommended [7]. However, there is no consensus amongst surgeons and clinical scientists on which treatment option is the best for treating PHF in the elderly [8,9,10,11,12,13,14]. It is even unclear what “best” means in this context: a significant increase in function measured by a clinical score, a reduction in pain, an increase in patient autonomy or quality of life? Several studies have analyzed non-operative and surgical techniques for treating elderly patients with PHF, most of them measuring functional outcomes—such as the range of motion (ROM)—or using clinical scores, such as the Constant–Murley score, the American Shoulder and Elbow Surgeons Shoulder Score (ASES) and others [15,16,17,18,19,20,21]. Many of these studies have either not found significant differences, only provided low levels of evidence, or reported conflicting results. However, even if significant differences are found in the functional scores between different treatment options, it does not necessarily mean that they exceed the minimal clinically important difference (MCID) [22].

The most important parameter from the patient’s perspective might, therefore, be the ability to regain quality of life (QoL). A PHF might impact the QoL on several different levels. Pain and a limited ROM can negatively impair the QoL on a physical level and can lead to a loss of independence and mobility. This can cause distress and may contribute to emotional and psychological challenges in older patients, further affecting their QoL. Reduced independence and mobility may also impact patients’ social lives, as it may be more difficult to participate in social activities or maintain relationships. Furthermore, assessing the QoL is also of clinical importance. It can improve patient-centered care and guide treatment decision making. However, although it constitutes such an important parameter for patients’ well-being, the QoL is vastly underrepresented in most studies evaluating PHF outcomes in older patients.

Several scores can be used to assess health-related QoL (HRQoL), but only a few studies have used them to evaluate elderly patients after PHF. In the context of PHF, the most commonly implemented scores that at least cover certain aspects of QoL are the Disabilities of Arm, Shoulder and Hand (DASH) score (or the shorter version quick DASH) [23,24], the Oxford Shoulder Score (OSS) [25], the 36-Item Short Form Survey (SF-36, or the shorter version SF-12) [26,27], the EuroQoL-5 Dimension (EQ-5D) [28] score, and the visual analogue scale (VAS) score to evaluate pain [29]. The VAS score is not a score to measure overall QoL, but as pain is an important factor for QoL and due to its easy and, therefore, frequent implementation, we included the VAS as a separate parameter for pain in this review. The DASH score covers certain aspects of QoL by asking questions about activities of daily living (ADL). However, it does not discriminate between the respective upper extremities. Hence, the DASH score is not a joint-specific questionnaire, but measures the function of both extremities as a whole [23]. The OSS and the SF-36 were not used as frequently in studies reporting on elderly patients with PHF. Therefore, in addition to the VAS, we focused on the EQ-5D score, since it is a standardized, widely used score specifically developed to assess HRQoL, which also shows strong validity in the elderly population [28,30].

The EQ-5D covers five dimensions to evaluate the HRQoL of a patient: (1) mobility, (2) self-care, (3) usual activities, (4) pain/discomfort, and (5) anxiety/depression. Five different levels are used to describe the dimensions further, from level 1 (no problems) to level 5 (severe problems), which can be converted into a score from 0–1 (0 = worst health status, 1 = best health status). The most commonly used score for the evaluation of pain is the subjective VAS, which ranges from 0 (no pain) to 10 (most imaginable pain). It is important to notice that the EQ-5D also includes a VAS; however, this VAS is not specific to pain only and ranges from 0 (worst possible health status) to 100 (best possible health status). The EQ-VAS is, therefore, not comparable to the VAS for pain and was not included in this review.

In this systematic review, we aimed to screen the current literature on the topic of QoL and pain in elderly patients after PHFs that were treated with the three most common methods [8,9,10]: (1) non-operative treatment (no-OP), (2) open-reduction and internal fixation using a locking plate (LPF), and (3) reverse total shoulder arthroplasty (RTSA). Our aim was to systematically describe and analyze the QoL and levels of experiences of pain in elderly patients after a PHF by means of VAS for pain and EQ-5D for QoL, and possibly reveal differences between three commonly used treatment methods.

## 2. Materials and Methods

### 2.1. Literature Search and Selection Criteria

**Figure 1:**
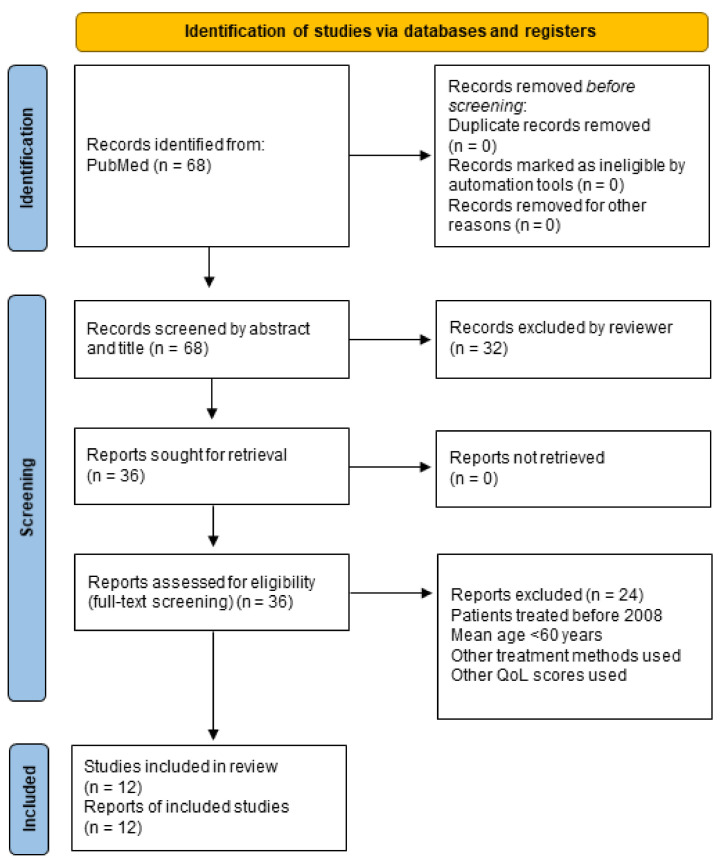
PRISMA flow diagram of search strategy and study selection.

A comprehensive search of the literature was performed in the bibliographic database PubMed (Bethesda (MD): National Library of Medicine (US)) from January 2023 up to April 2023 by two independent researchers. The following research terms were used: “proximal humer* AND fracture AND (arthroplasty OR plate OR fixation OR non-operative OR conservative) AND (VAS OR EQ5D OR “quality of life”)”. The following filters were used: publication year 2010–2023, article type: clinical study, clinical trial, comparative study, controlled clinical trial, multicenter study, randomized controlled trial. Comparative or descriptive studies including RCT, describing the quality of life or pain after treatment of a PHF either non-operatively (non-OP) or with LPF or RTSA were included. Only articles including patients aged 60 years or older treated after 2008 were considered. Studies with a sole follow-up (FU) time of less than 12 months were also excluded. As not enough studies that met our other inclusion criteria used the OSS and SF-36, we only included studies using the EQ-5D (ranging from 0, the worst possible health status, to 1, the best possible health status) to assess QoL, or the visual analogue scale (VAS) to describe pain on a scale from 0–10 (0 = no pain, 10 = most imaginable pain). The DASH score was also excluded, since it does not discriminate between an injured and healthy shoulder. Articles were screened by their title and abstract and duplicates were removed. Articles that initially met all the criteria were then further evaluated by a full-text review to collect data. The review was prepared according to the Preferred Reporting Items for Systematic Reviews (PRISMA) statement without a registration protocol (see Figure 1). 

### 2.2. Risk-of-Bias Assessment

**Figure 2:**
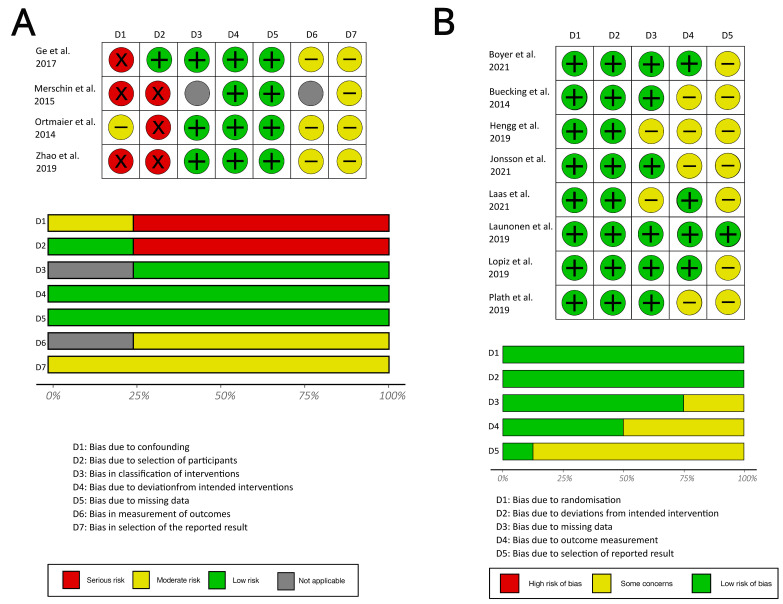
Overview of the risk-of-bias assessment for the included studies. (**A**) ROBINS-I: risk-of-bias assessment for included non-randomized trials. (**B**) ROB 2: risk-of-bias assessment for included randomized controlled trials [21,33,34,35,36,37,38,39,40,41,42,43].

Both RCT and non-randomized trials were included in the analysis. The risk of bias was, therefore, assessed by two collaborating reviewers using two different types of analysis tools (see Figure 2), the ROB 2 tool for assessing RCTs and the ROBINS-I tool for assessing non-randomized trials [31,32].

### 2.3. Statistical Analysis

**Figure 3:**
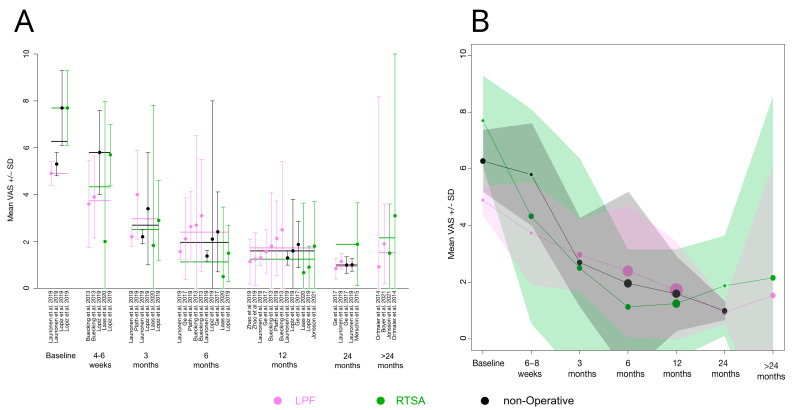
Overview of all reported and calculated VAS scores of the included studies at specific FU times. (**A**) Reported mean VAS scores (mean ± SD) of all included studies for each FU and treatment represented by a horizontal line in the indicated color (violet: LPF; green: RTSA; black: non-operative treatment). (**B**) The overall calculated mean VAS score (mean ± SD) for each FU. Overall SDs were conservatively approximated based on the reported SDs of the single studies [21,33,34,35,36,37,38,39,40,41,42,43].

**Figure 4:**
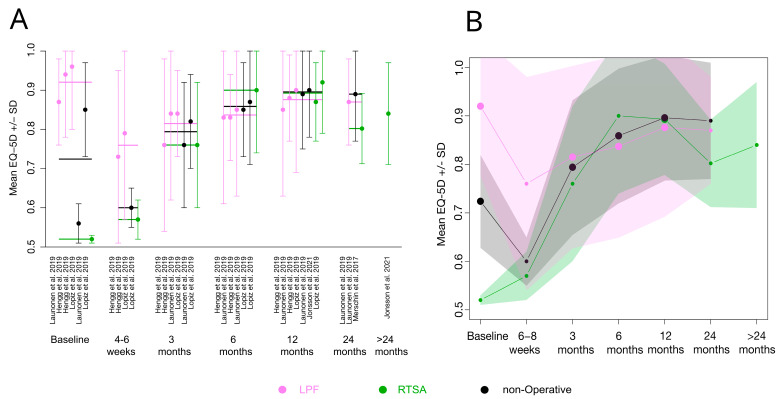
Overview of all reported EQ-5D indices of the included studies at different FU times. (**A**) Reported mean EQ-5D indices (mean ± SD) of all included studies for each FU and treatment represented by a horizontal line in the indicated color (violet: LPF; green: RTSA; black: non-operative treatment). (**B**) The overall calculated mean EQ-5D indices (mean ± SD) for each FU. Overall SDs were conservatively approximated based on the reported SDs of the single studies [21,33,34,35,36,37,38,39,40,41,42,43].

Of primary interest for the study at hand, the mean VAS and mean EQ-5D scores after LPF, RTSA, or the non-operative treatment of PHF were analyzed. We examined 12 studies that assessed the VAS or EQ-5D after PHF in elderly patients treated with LPF, RTSA or the non-operative treatment. Eleven studies reported the VAS score and five studies reported the EQ-5D score at one or more of the following seven FU times: baseline, 4–6 weeks, 3 months, 6 months, 12 months, 24 months or >24 months. Two of the eleven studies compared two different LPF variants or different surgical approaches of LPF, respectively [33,34]. Data were collected from both reported groups and were treated as separate LPF groups, i.e., it is possible that one study yielded more than one mean VAS/EQ-5D value. Table 1 shows the reported VAS score, EQ5D score, sample size, age in years, and proportion of women for all the individual studies and data FU times. Categorical variables were described as absolute and relative numbers; metric variables were described as mean ± standard deviation (SD). If VAS values were reported as median values, they were converted to an approximate mean using the method of Hozo et al. [44] when reported with the minimum, maximum and sample size (Table 1, cases are marked with “γ”), or using the method of Wan et al. [45], when reported with the 1st and 3rd percentiles and sample size (Table 1, cases are marked with “τ”). In some cases, standard deviations (SD) had to be computed based on reported confidence intervals or standard errors. When only the range was given as a dispersion parameter (Table 1, cases are marked with “£”), a strict approximation to the standard deviation (SD) was calculated by taking the range as a 95% confidence interval. All available VAS and EQ-5D scores at the seven times of FU were averaged and plotted over time (Figure 3B and Figure 4B). Standard deviations of the mean values were conservatively estimated based on the SD of the individual studies. Since the data situation was insufficient for a more in-depth analysis, such as a meta-analysis or a network analysis, the development of the VAS and the EQ-5D score was only examined descriptively. It should be noted that the scores are paired across time periods and are, therefore, highly interdependent, as different times of data collection and different treatment groups from the same studies were included. However, not all time periods are covered by all studies. In addition, some treatment variants are combined into one generic term. Since no treatment effect results were used, but rather reported individual scores of sometimes only one treatment, a further heterogeneity analysis of the included studies was not considered useful. This descriptive analysis was, thus, intended to serve as a mere overview of the existing literature and has no claim to general validity. All calculations were carried out with R Studio Version 1.3.53 and R Version 4.1.2.54, R foundation, Vienna, Austria.

## 3. Results

### 3.1. Literature Search Strategy and Study Selection

We identified 68 references with the described search strategy. After the screening of titles and abstracts, we retrieved 36 articles for full-text review. Subsequently, 12 studies with 449 LPF, 146 RTSA and 117 non-OP patients at baseline met our inclusion criteria, as outlined in Figure 1. The included studies featured only one direct comparison between LPF and RTSA, one comparison between RTSA and non-OP and two comparisons between LPF and non-OP. The rest of the studies were either observational studies (one study), compared LPF/RTSA/non-OP to other treatment methods (hemiarthroplasty or intramedullary nail; four studies) or compared different surgical approaches with LPF (such as with or without screw augmentation, fibular allograft, etc.; three studies). Eleven studies reported the VAS and five studies reported the EQ-5D score. A total of 712 patients at baseline were included (78% female sex, mean age of 75.2 years). A full overview of the included studies can be found in Table 1.

### 3.2. Risk of Bias Analysis

Eight of the twelve studies were RCTs, two studies were retrospectively evaluated comparative studies of two interventions, one study was a retrospectively evaluated descriptive study of one surgical intervention and one study was a non-randomized prospective study comparing a surgical intervention and non-operative treatment. Thus, both randomized controlled trials (RCT) and non-randomized studies of interventions (NRSI) were included in the risk-of-bias analysis. The risk of bias was assessed using two different types of analysis tools, the ROB 2 tool [32] for assessing RCTs and the ROBINS-I tool [31] for assessing non-randomized trials. For NRSIs, the risk of bias due to confounding was rated as “moderate” if confounding could not be excluded in principle but cohort matching had taken place. If the cohorts were not matched, the bias due to confounding was classified as “serious risk”, even if the baseline tables did not show a difference between the treatment groups (Figure 2A, D1). Bias originating from outcome measurements (Figure 2A, D4 and D6) was rated as “some concerns” or “moderate risk”, if the assessor of the outcome measurement was not explicitly stated to be blinded. Bias due to selection of reported result (Figure 2A, D7, and Figure 2B, D5) was rated as “some concerns” or “moderate risk”, if no previously defined study protocol was found. The risk of bias in the selection of participants for the intervention was rated as serious for studies based on retrospective data analyzes (Figure 2A, D2). Possible biases concerning treatment differences were not applicable in studies investigating only one treatment.

### 3.3. VAS Score for Pain Evaluation

The VAS score for pain was reported by 11 studies. The majority of studies used the VAS on a scale from 0–10, with 0 being no pain and 10 being the most imaginable pain. Two studies (Jonsson et al. (2021) [38], and Launonen et al. (2019) [40]) used a scale from 0–100 and results were, therefore, divided by 10 to be included in the overall calculation. The reported VAS at 12-month FU ranged from 0.7 to 2.5 (Table 1). Values categorized in the FU of >24 months ranged from a mean FU time of 31.8 months to 66 months (Table 1). Three studies reported a significant difference in the VAS score. Ortmaier et al. (2015) [42] reported significantly less pain in patients at the last FU (with a minimum of 12 months and a mean of more than 30 months) that were treated with LPF compared to RTSA (VAS 0.9 vs. 3.1; *p* < 0.01). Lopiz et al. (2019) [21], on the other hand, reported significantly less pain at 12-month FU in patients that were treated with RTSA compared to non-operatively treated patients (VAS 0.9 vs. 1.6; *p* = 0.01). Boyer et al. (2021) [35] reported significantly higher pain levels at a mean FU of 66 months in LPF patients than in patients treated with intramedullary nails (VAS 1.9 vs. 0.9; *p* = 0.001). All other studies did not find a significant difference in the VAS score for RTSA, LPF or non-OP treatment. 

All reported mean VAS scores of each study for different FU times are shown in Figure 3A. The calculated overall mean VAS score across all studies showed a decreasing tendency for all treatments with increasing FU time up to 12 months after PHF (Figure 3B, Table A1). We observed a slight increase in the VAS score at a FU of 24 months or more in RTSA patients and a slight increase in the VAS score at a FU of more than 24 months in LPF patients. There were no data available for non-operatively treated patients at a FU of more than 24 months in the included studies. However, as mentioned above, mean FU times of more than 24 months varied greatly in the included studies and the internally dependent data structure remains.

### 3.4. EQ-5D for Evaluation of Health-Related QoL

The EQ-5D score was reported by five studies. The reported EQ-5D index ranged from 0.85 to 0.92 (Table 1). None of the studies reported any significant differences across the groups. Overall calculated EQ-5D indices showed an increasing trend after 6–8 weeks FU, but did not differ significantly between the three treatments (Figure 4, Table A2). Furthermore, baseline values differed significantly in the three treatment groups. It should be noted that a generally valid statement is not possible on the basis of the available data.

## 4. Discussion

In this systematic review, we aimed to analyze whether there were differences in the QoL or pain outcomes in elderly patients (>60 years old) with a PHF after they received non-operative treatment or surgical treatment using LPF or RTSA. As there is an abundance of different score systems to assess QoL or aspects of QoL, we focused on the EQ-5D, as it is standardized, widely used, specifically developed to assess HRQoL and also shows strong validity in the elderly population [28,30]. In addition, we analyzed the VAS, since pain is a major factor influencing QoL. However, during our research of the literature, it became clear that most studies on the treatment of PHF in the elderly do not focus on QoL but rather on functional outcomes (measured for instance by the range of motion or scores such as the Constant–Murley score). Hence, the main result of this review is that there is not enough evidence to conclude whether non-operative treatment or surgical treatment with LPF or RTSA in elderly patients with a PHF is better regarding the QoL and/or pain outcome. A low HRQoL, for instance due to reduced independence and mobility, can result in a reduced overall life satisfaction and happiness, which has been shown to impact overall survival and mortality [46]. Furthermore, a reduced HRQoL, especially in the elderly, can be a huge financial burden on the health system, as reduced psychological well-being can impact treatment success, as has been shown for hip fractures [47], which, of course, is also of clinical relevance. Consequently, raising awareness of the lack of QoL data in the treatment of PHF in the elderly is the main purpose of this review. Future clinical trials should aim to overcome this deficit and should urgently include measurements of QoL and pain in addition to functional scores and assessments of cognitive status, previous frailty and the risk of falling. Only then might we be able to offer individualized, patient-centered care to the elderly with PHF.

Only one study that met our inclusion criteria directly compared LPF with RTSA. Ortmaier et al. [42] reported a significantly reduced VAS score in LPF patients (VAS 0.9) compared to RTSA patients (VAS 3.1). However, with only 25 patients in each group, the sample size was rather low, and due to the retrospective nature of the analysis, selection bias cannot be excluded. In contrast, Lopiz et al. [21] reported a significantly reduced VAS score in RTSA patients (VAS 0.9) compared to patients treated non-operatively (VAS 1.6). The overall calculation of the mean VAS score across all included studies was, therefore, fairly similar, with scores ranging between 1.25 for RTSA-treated patients, 1.60 for non-operatively treated patients and 1.73 in the LPF group at 1 year FU. The minimally clinically important difference (MCID) of VAS, based on data from adult rheumatology patients, has been suggested to be a reduction by 1.37 [48]. Although pain is an important factor for QoL, it is well known that it is a very subjective experience and might be influenced by several factors such as age, comorbidities and psychological well-being.

Five of the included studies reported on the EQ-5D in elderly patients with PHF. None of them reported any significant differences, with EQ-5D indices ranging between 0.80 and 0.90 in the three treatment groups at one year FU. The overall calculation of the mean EQ-5D indices across all included studies were also relatively similar, ranging between 0.88 for LPF patients, 0.90 for non-operatively treated patients and 0.92 for RTSA patients at one year FU. Although all three groups received good scores in the EQ-5D, it would have been interesting to see if there were differences across the five dimensions of the EQ-5D score. The MCID of the EQ-5D index for PHF, assessed in relation to a DASH MCID of 10, has been suggested to be an increase of 0.12 [28]. 

Our findings are in line with the previously published literature. Handoll et al. [16] published a meta-analysis in 2022 on treatment methods for PHFs in adults. They reported, with high-certainty evidence, that there is no clinically important difference in QoL at one- and two-year FU for the EQ-5D between surgical and non-operative treatments (mean difference for one year FU: 0.01; 95% CI −0.02 to 0.04; six studies; 502 participants). The same was true for the VAS score (VAS measured here from 0–100; mean difference for one year FU: −3.64, 95% CI −9.97 to 2.51; two studies, 62 participants), the OSS, the DASH and the SF-12 at 12 months FU. However, it is important to mention that the treatment approach is certainly not the only factor that can influence the QoL and levels of experienced pain in the context of PHF. The fracture severity, comorbidities, functional outcome, previous frailty, cognitive status, physical therapy and rehabilitation, as well as social support, might also influence both the functional outcome and the QoL, including pain. For instance, Henkelmann et al. [49] reported that the shoulder-specific outcome after PHF influences the medium-term overall QoL, as measured by the EQ-VAS.

It should be noted that one of the major criticisms brought against the use of generic HRQoL questionnaires such as the EQ-5D is that these instruments may not be sensitive enough to detect specific changes in a particular disease of interest. Therefore, assessments of QoL should always be implemented together with functional measurements. Furthermore, they might be insensitive to changes over time and treatment [50]. Disease-specific QoL scores are available for some musculoskeletal diseases, for instance, for osteoporosis-related or rheumatoid arthritis-related QoL. However, for osteoporosis alone, there are currently no less than six disease-specific QoL scores (Qualeffo-4148 [51], questionnaire QoL in Osteoporosis [QUALIOST] [52], osteoporosis assessment questionnaire [OPAQ] [53], osteoporosis QoL questionnaire [OQLQ] [54], osteoporosis functional disability questionnaire [OFDQ] [55] and osteoporosis-targeted QoL questionnaire [OPTQoL] [56]). Thus, it is difficult, if not impossible, to compare and/or meta-analyze the results of studies that used different score systems.

### Limitations

This systematic review has several limitations. First, the results of the current review are based on relatively small trials, which demonstrate ‘no evidence of an effect’ rather than ‘evidence of no effect’. Moreover, only descriptive analyses were possible due to small numbers of studies and lack of direct comparisons. The descriptive analysis included all reported FU periods, although not all periods were covered by all studies, and some treatment options were grouped under one umbrella term. This resulted in highly interdependent data and gave more weight to studies reporting many FU periods.

## 5. Conclusions

In conclusion, the current literature suggests that there are no clinically important differences between QoL or pain in elderly patients with PHF after non-operative treatment or surgical treatment with LPF or RTSA. However, the number of studies and level of evidence is rather low and further trials are urgently needed. Individual treatment decisions should consider many different factors in addition to the prospective QoL, pain and functional outcome, such as complication and major-adverse-event rates [11,57], mortality and gender [4], as well as cognitive status, previous frailty and the risk of falling. Further randomized controlled trials that also include measurements of QoL in addition to functional outcome measurements are, therefore, necessary to determine the “best” individual treatment option for PHF for elderly patients.

## Figures and Tables

**Table 1:** Overview of the included studies and reported VAS and EQ-5D scores. Data are given as mean (±SD). Footnotes: *£*—ranges where interpreted as 95% confidence interval in order to obtain a conservative approximation for the SD; τ—mean is approximated based on reported median, 1st and 3rd quartile;γ—mean is approximated based on reported median and range; ¥—VAS mean is averaged based on separately reported results on 3-part and 4-part fractures, and VAS SD is approximated conservatively; ●—marked the treatment variant in the study. For more details, see Section 2.3. Abbreviations: SD, standard deviation. RCT, randomized controlled trial. n.r., not reported.

Study					Baseline			Follow-Up			
Study Design		Treatment			Sample Size (N)	Age in Years	Female N (%)	Follow-Up in Months	Sample Size (N)	VAS	EQ-5D
		RTSA	LPF	Non-OP							
Boyer et al., 2021 [35]	RCT		●		50	77 (±28.1)	29 (69%)	66 (±13.5)	42 (100%)	1.9 (±1.7)	n.r.
Buecking et al., 2014 [34]	RCT		●		60	69 (±19.4)	48 (80%)	1.5	48 (80%)	3.6 (±1.9)	n.r.
								6	48 (80%)	2.7 (±3.8)	n.r.
								12	48 (80%)	1.8 (±2.3)	n.r.
			●		60	67 (±40.7)	44 (73%)	1.5	42 (70%)	3.9 (±1.8)	n.r.
								6	42 (70%)	3.1 (±2.4)	n.r.
								12	42 (70%)	2.5 (±2.9)	n.r.
Ge et al., 2017 [36]	Non-RCT		●		69	75 (±8.5)	45 (65%)	6	69 (100%)	$2.1\;(\pm {1.8)}^{¥}$	n.r.
								12	69 (100%)	$1.6\;(\pm {0.9)}^{¥}$	n.r.
								24	69 (100%)	0.8 $(\pm {0.4)}^{¥}$	n.r.
				●	43	74 (±7.7)	34 (79%)	6	43 (100%)	$2.4\;(\pm {1.7)}^{¥}$	n.r.
								12	43 (100%)	$1.9\;(\pm {1.0)}^{¥}$	n.r.
								24	43 (100%)	$1.0\;(\pm {0.4)}^{¥}$	n.r.
Hengg et al., 2021 [37]	RCT		●		34	76 (±6.2)	29 (85%)	0	27 (79%)	n.r.	0.96 (±0.16)
								3	27 (79%)	n.r.	0.84 (±0.22)
								6	27 (79%)	n.r.	0.85 (±0.22)
								12	27 (79%)	n.r.	0.90 (±0.21)
			●		33	78 (±7.4)	26 (79%)	0	30 (91%)	n.r.	0.94 (±0.16)
								3	30 (91%)	n.r.	0.76 (±0.22)
								6	30 (91%)	n.r.	0.83 (±0.22)
								12	30 (91%)	n.r.	0.85 (±0.22)
Jonsson et al., 2021 [38]	RCT	●			41	80 (±4.5)	39 (95%)	12	36 (88%)	1.8 (±1.9)	0.87 (±0.10)
								≥ 24	36 (88%)	1.5 (±2.1)	0.84 (±0.13)
Laas et al., 2020 [39]	RCT	●			17	75 (±26.3)	9 (52%)	1.5	17 (100%)	$2.0^{\tau }$ (±6.0)	n.r.
								3	17 (100%)	$1.8^{\tau }$ (±6.0)	n.r.
								6	17 (100%)	$0.5^{\tau }$ (±3.0)	n.r.
								12	17 (100%)	$0.7^{\tau }$ (±3.0)	n.r.
Launonen et al., 2019 [40]	RCT		●		44	72 (±7.4)	41 (95%)	0	44 (100%)	4.9 (±0.5)	0.87 (±0.13)
								3	40 (91%)	2.2 (±0.4)	0.84(±0.13)
								6	37 (84%)	1.6 (±0.3)	0.83 (±0.12)
								12	33 (75%)	1.3 (±0.3)	0.88 (±0.11)
								24	33 (75%)	1.2 (±0.3)	0.87 (±0.11)
				●	44	73 (±42.8)	39 (87%)	0	44 (100%)	5.3 (±0.5)	0.85 (±0.13)
								3	42 (95%)	2.2 (±0.3)	0.82 (±0.13)
								6	40 (91%)	1.4 (±0.2)	0.85 (±0.13)
								12	39 (89%)	1.3 (±0.3)	0.90 (±0.12)
								24	39 (89%)	1.0 (±0.3)	0.89 (±0.12)
Lopiz et al., 2019 [21]	RCT	●			29	82 (±3.2)	25 (86%)	0	29 (100%)	7.7 (±1.6)	0.52 (±0.01)
								1	29 (100%)	5.7 (±1.3)	0.57 (±0.05)
								3	29 (100%)	2.9 (±1.7)	0.76 (±0.16)
								6	29 (100%)	1.5 (±1.2)	0.90 (±0.16)
								12	29 (100%)	0.9 (±0.9)	0.92 (±0.13)
				●	30	85 (±4.8)	26 (87%)	0	30 (100%)	7.7 (±1.6)	0.56 (±0.05)
								1	30 (100%)	5.8 (±1.8)	0.60 (±0.05)
								3	30 (100%)	3.4 (±2.4)	0.76 (±0.16)
								6	30 (100%)	2.1 (±5.9)	0.87 (±0.16)
								12	30 (100%)	1.6 (±2.2)	0.89 (±0.14)
Merschin et al., 2015 [41]	Non-RCT	●			34	80 (±6.7)	28 (85%)	23 (±10.7)	33 (97%)	1.9 (±1.8)	0.8 (±0.09)
Ortmaier et al., 2014 [42]	Non-RCT	●			25	$73\;(\pm 29.1{)\;}^{£}$	22 (88%)	$32.7\;(\pm 25.4{)\;}^{£}$	25 (100%)	$3.1\;{\left(\pm 9.7\right)}^{£}$	n.r.
			●		25	73${\left(\pm 24.2\right)}^{£}$	22 (88%)	$31.8(\pm 29.1{)\;}^{£}$	25 (100%)	0.9 ${\left(\pm 7.3\right)}^{£}$	n.r.
Plath et al., 2019 [43]	RCT		●		32	$77{\left(\pm 41.3\right)}^{£}$	25 (78%)	3	30 (94%)	${4.0\;}^{\gamma }$ (±1.4)	n.r.
								6	29 (91%)	${2.6\;}^{\gamma }$ (±1.5)	n.r.
								12	28 (88%)	$2.1^{\;\gamma }$ (±1.6)	n.r.
Zhao et al., 2019 [33]	Non-RCT		●		21	69 (±7.2)	9 (43%)	12	21 (100%)	1.3 (±1.1)	n.r.
			●		21	69 (±6.3)	12 (57%)	12	21 (100%)	1.1 (±1.0)	n.r.
Overall		5	11	3	712	75	552 (78%)				

## Data Availability

No new data were created or analyzed in this study. Data sharing is not applicable to this article.

## Appendix A

**Table A1:** Calculated mean VAS scores of all included studies for different FU times. Data are given as mean ± SD and are visualized in Figure 3B. For LPF, the number of studies differed from the number of yielded values (n), as two studies reported on different LPF variants or different surgical approaches to LPF, respectively [33,34].

VAS		FU Time after Treatment Start						
		Baseline	4–6 Weeks	3 Months	6 Months	1 Year	2 Years	>2 Years
LPF	number of studies	1	1	2	4	5	2	2
	number of values (n)	1	2	2	5	7	2	2
	VAS	4.90	3.74	2.97	2.40	1.73	0.94	1.53
	SD	0.50	1.82	1.28	2.33	1.73	0.41	4.63
RTSA	number of studies	1	2	2	2	3	1	2
	number of values (n)	1	2	2	2	3	1	2
	VAS	7.70	4.33	2.51	1.13	1.25	1.88	2.16
	SD	1.60	3.78	3.88	2.04	1.92	1.77	6.42
Non-OP	number of studies	2	1	2	3	3	2	0
	number of values (n)	2	1	2	3	3	2	0
	VAS	6.27	5.80	2.70	1.96	1.60	0.99	-
	SD	1.09	1.80	1.57	3.22	1.31	0.32	-

**Table A2:** Calculated mean EQ-5D indices of all included studies at different FU times. Data are given as mean ± SD and are visualized in Figure 4B. For LPF, the number of studies differed from the number of yielded values (n), as two studies reported on different LPF variants or different surgical approaches to LPF, respectively [33,34].

EQ-5D		FU Time after Treatment Start						
		Baseline	4–6 Weeks	3 Months	6 Months	1 Year	2 Years	>2 Years
LPF	number of studies	2	1	2	2	2	1	0
	number of values (n)	3	2	3	3	3	1	0
	EQ-5D	0.92	0.76	0.82	0.84	0.88	0.87	-
	SD	0.14	0.22	0.19	0.19	0.18	0.11	-
RTSA	number of studies	1	2	1	1	1	1	1
	number of values (n)	1	2	1	1	1	1	1
	EQ-5D	0.52	0.73	0.76	0.90	0.92	0.80	0.84
	SD	0.01	0.08	0.16	0.16	0.13	0.09	0.13
Non-OP	number of studies	2	1	2	2	2	1	0
	number of values (n)	2	1	2	2	2	1	0
	EQ-5D	0.72	0.60	0.79	0.86	0.90	0.89	-
	SD	0.10	0.05	0.14	0.14	0.13	0.12	-
